# Poly(vinyl alcohol)/Silk Fibroin/Ag-NPs Composite Nanofibers as a Substrate for MG-63 Cells’ Growth

**DOI:** 10.3390/polym15081838

**Published:** 2023-04-11

**Authors:** Monica L. Mejía Suaza, Jennifer C. Leos Rivera, Maria C. Rodríguez Padilla, Maria E. Moncada Acevedo, Claudia P. Ossa Orozco, Diana G. Zarate Triviño

**Affiliations:** 1 Advanced Materials and Energy (MATyER) Research Group, Faculty of Engineering, Metropolitan Technological Institute (ITM), Medellin 050012, Colombia; 2 Laboratory of Immunology and Virology, Faculty of Biological Sciences, Autonomous University of Nuevo Leon, Monterrey 64000, Mexico; 3 Biomaterials Research Group, Faculty of Engineering, University of Antioquia, Medellin 050010, Colombia

**Keywords:** silver nanoparticles, nanofibers, polyvinyl alcohol (PVA), silk fibroin, electrospinning, bone tissue engineering

## Abstract

Nanofiber scaffolds of polyvinyl alcohol, silk fibroin from *Bombyx mori* cocoons, and silver nanoparticles were developed as a substrate for MG-63 growth. The fiber morphology, mechanical properties, thermal degradation, chemical composition, and water contact angle were investigated. In vitro tests were performed by the cell viability MTS test of MG-63 cells on electrospun PVA scaffolds, mineralization was analyzed by alizarin red, and the alkaline phosphatase (ALP) assay was evaluated. At higher PVA concentrations, Young’s modulus (E) increased. The addition of fibroin and silver nanoparticles improved the thermal stability of PVA scaffolds. FTIR spectra indicated characteristic absorption peaks related to the chemical structures of PVA, fibroin, and Ag-NPs, demonstrating good interactions between them. The contact angle of the PVA scaffolds decreased with the incorporation of fibroin and showed hydrophilic characteristics. In all concentrations, MG-63 cells on PVA/fibroin/Ag-NPs scaffolds had higher cell viability than PVA pristine. On day ten of culture, PVA18/SF/Ag-NPs showed the highest mineralization, observed by the alizarin red test. PVA10/SF/Ag-NPs presented the highest alkaline phosphatase activity after an incubation time of 37 h. The achievements indicate the potential of the nanofibers of PVA18/SF/Ag-NPs as a possible substitute for bone tissue engineering (BTE).

## 1. Introduction

The bone is a rigid tissue that supports the body’s weight, supplies blood cells, stores minerals, and protects internal organs [1,2]. Under normal physiological circumstances, the bone has the ability to regenerate and mend itself. However, severe cases of bone abnormalities, such as fractures, tumors, and skeletal illnesses (such as osteoporosis), impair the ability of the bone to mend itself naturally, requiring the use of bone substitutes [1,2,3]. Tissue engineering, which employs a variety of biomaterials and techniques, has become an alternative for repairing bone-critical defects [4]. An ideal bone scaffold regulates cell adhesion, growth, and proliferation, and it should be biocompatible, biodegradable, mechanically robust, and mimic the extracellular matrix (ECM) [1,4,5].

For bone tissue engineering (BTE), various techniques have been employed to create nanofiber scaffolds, including gas foaming, solvent casting, freeze-drying, 3D printing, and electrospinning [6].

The morphology of micro/nanofibers produced by electrospinning has attracted attention because it closely resembles the extracellular matrix (EMC) found in living things. Scaffolds fabricated by electrospinning have advantages as a structure that is very porous, has a high surface area-to-volume ratio, and includes tiny fibrous interconnects. These characteristics encourage cell adhesion, proliferation, and migration to form new bone tissue [7,8]. In electrospinning, aligned or random scaffolds are produced from a polymer solution to which a high voltage is applied. The solutions may consist of synthetic or natural polymers [9,10].

The synthetic polymer polyvinyl alcohol (PVA) is created by hydrolyzing polyvinyl acetate. PVA offers beneficial qualities such as non-toxicity, non-carcinogenicity, biocompatibility, biodegradability, transparency, water solubility, hydrophilicity, good swelling capabilities in aqueous mediums, good chemical, thermal stability, and mechanical strength connected to good flexibility [11]. Due to the many polar alcohol groups creating hydrogen bonds, PVA dissolves readily in water [12]. This polymer is suited for creating continuous, bead-free nanofibers because of its electroconductivity [9,11,13]. Due to PVA’s anabolic effect on bone growth, bone tissue engineering has been investigated [14].

Obtainable from the cocoons of silkworms, fibroin (SF) is a naturally occurring fibrous protein. Numerous biomaterials have been created using SF (e.g., gels, sponges, scaffolds, and films) [15,16].

Fibroin has attracted increased attention in recent years for its applications in bone tissue engineering because it possesses high biocompatibility, low immunogenicity, microbial resistance, a low degradation rate, high oxygen and water vapor permeability, and structural integrity [1,7,8,9,11]. However, fibroin is brittle in the dry state, so it can be easily broken, but its mechanical properties can be improved by combining it with other polymers [1].

Fibroin works well when mixed with PVA. Fibroin contains amide and hydroxyl groups, which are potentially miscible with PVA through the formation of hydrogen bonds [17], while PVA may enhance the mechanical flexibility of SF [18].

For the preparation of fibroin and PVA solutions for electrospinning, the use of organic solvents such as hexafluoroisopropanol (HFIP), trifluoroacetic acid (TFA), dichloromethane (DCM), and formic acid should ideally be avoided as they can be toxic for cell culture, and water is recommended for biomedical applications [18,19].

PVA and fibroin scaffolds have been explored with silver nanoparticles (Ag-NPs) to enhance the antibacterial properties against *E. coli* and *S. aureus* [20,21]. Ag-NPs are efficient even at low concentrations and have a diameter range of 1 to 100 nm. The large relative surface area of scaffolds expands the number of microorganisms (bacteria and fungi) with which they come into contact [20,21].

However, the PVA/fibroin scaffold has not been extensively studied for bone tissue engineering. Rosuvastatin (RSV) (50 mg), PVA (1 g), and *Bombyx mori* fibroin at a concentration of 13% wt were blended together with formic acid as a solvent by M. Kalani et al. (2019), and the scaffold was cultivated using human adipose-derived stem cells (MG63). This promoted cell division and supported osteogenic differentiation [1]. T. Kobori et al. (2007) utilized water as the solvent, and PVA at a concentration of 10% wt, and *Antheraea mylitta* fibroin at a concentration of 2–4% wt. Human osteoblast cells were used to cultivate the scaffold, and they demonstrated osteoconductivity [22].

In our previous study, we prepared, for the first time, a composite scaffold of polyvinyl alcohol (PVA)/*Bombyx mori* fibroin (SF)/silver nanoparticles (Ag-NPs) as a possible substrate for bone tissue engineering that exhibited desirable properties, such as porosity and a high surface area-to-volume ratio [23]. However, biological evaluation with MG-63 cells has not been performed so far. In this article, we further elaborate on cell viability (MTS), alizarin red (AR), and alkaline phosphatase (ALP) assays to determine their application in bone tissue engineering.

## 2. Materials and Methods

### 2.1. Materials

Silk cocoons from *Bombyx mori* silkworms were purchased in Corseda (Popayan, Cauca, Colombia). Sodium carbonate (Na_2_CO_3_), cellulose dialysis tube (12 kDa, MWCO), calcium chloride (CaCl_2_), distillate water, and absolute ethanol (99.7%) were obtained from Merck (Darmstadt, Germany). Polyvinyl alcohol (medium molecular weight, Mw = 89,000 to 98,000 Da), 99% hydrolyzed, was provided by Sigma-Aldrich (Saint Louis, MO, USA). Silver nitrate (AgNO_3_) at 100% purity and trisodium citrate dihydrate at 99% purity were both obtained from PanReac (Barcelona, Spain). All the reagents were of analytical grade.

### 2.2. Fibroin Extraction

*Bombyx mori* silk cocoons were cut into small pieces (5 g) and degummed in 0.5% (*w*/*v*) of sodium carbonate (Na_2_CO_3_) aqueous solution (500 mL) for 1 h at 100 °C, and then washed with distilled water several times to remove sericin. The degummed fibroin was dried overnight at 37 °C before being dissolved in a molar ratio of (1:2:8) calcium chloride, ethanol, and water at 60 °C for 1 h to simplify the fibroin structure. The fibroin solution was dialyzed with a food cellulose membrane against deionized water for 3 days [24], with water replacement every 2–6 h to remove salt residue. The solution was centrifuged 2 times at 3000 rpm for 10 min to remove silk aggregates as well as debris from the original silk. Then, the fibroin solution was filtered to remove impurities. The fibroin solution was stored at 5 °C prior to use. The final concentration of the fibroin aqueous solution was about 2% wt.

### 2.3. Ag-NPs’ Synthesis

The silver nanoparticles were synthesized by chemical reduction, making modifications to the protocol implemented by Cuervo-Osorio et al. in 2020 [25]. Distilled water was heated to a temperature of 100 °C, and then 0.1 M AgNO_3_ was added as a precursor agent and stirred for 10 min. Then, 1% wt trisodium citrate dihydrate was slowly added as a reducing agent, maintaining stirring until the solution turned yellow and was allowed to cool to room temperature.

### 2.4. Preparation of Electrospinning Solutions

PVA pristine solutions were prepared at 10% wt, 15% wt, and 18% wt, dissolved in water. PVA/SF/Ag-NPs solutions were prepared with distilled water as a solvent in a concentration of PVA/SF (90:10) (*v*/*v*) at a concentration of PVA of 10% wt, 15% wt, and 18% wt, all samples with fibroin (2% wt) and silver nanoparticles (0.5% wt), in which it complies with its antibacterial characteristics, without compromising cell behavior [26,27]. They were mixed together and stirred for 1 h at 80 °C, and the samples were labeled as PVA10/SF/Ag-NPs, PVA15/SF/Ag-NPs, and PVA18/SF/Ag-NPs, as shown in Table 1.

### 2.5. Preparation of Nanofiber Scaffolds

The electrospinning process was performed on FLUIDNATEKTM LE 100 equipment at room temperature with a relative humidity of 42%. The solutions were loaded in a 5 mL plastic syringe. The distance from the injector to the collector was 20 cm, the voltage was 18 kV, and the flow rate was 0.3 mL/h. As a collector, a 15 × 15 cm^2^ piece of aluminum foil was used. The PVA pristine and PVA/SF/Ag-NPs composite scaffolds were vacuum-dried in an oven for 48 h.

### 2.6. Characterization of Silver Nanoparticles (Ag-NPs)

#### 2.6.1. Formation of Ag-NPs (UV-Vis) Spectrometry

The spectral UV-visible range of the Ag-NPs’ suspension was obtained with a 3200 PC MAPADA UV-vis spectrometer in absorbance mode over the range of 300–600 nm.

#### 2.6.2. Average Ag-NPs’ Size (DLS)

Silver nanoparticles’ average size, polydispersity index, and Z-potential were determined using the dynamic light scattering (DLS) technique in the Nanoplus HD equipment at a wavelength of 632.8 nm and a detection angle of 90° at 25 °C. The measurements were performed in triplicate.

#### 2.6.3. Morphology and Size of Ag-NPs (TEM)

The size, distribution, and morphology of the silver nanoparticles (Ag-NPs) were determined by transmission electron microscopy (TEM) using the equipment FEI TECNAI G2, F20 S-TWIN. As a pretreatment, a drop of the colloidal suspension of Ag-NPs was deposited on a copper grid and allowed to dry under a laminar flow hood. Then, the micrographs were taken at a voltage of 200 kV and a magnification of 10× up to 30,000× and analyzed in Image J software. The average size of silver nanoparticles was determined by taking 20 measurements. The presence of silver was determined using the TEM’s EDX.

### 2.7. Characterization of Nanofibers’ Scaffolds

#### 2.7.1. Morphology of Electrospun Fibers (FE-SEM)

Morphological analyses were carried out using a field-emission microscope (FE-SEM) (JEOL JSM-7100F). The samples of pristine PVA and PVA/SF/Ag-NPs composite scaffolds were coated with gold using a sputtering Quorum Q300T for 10 min at 10 mA to supply electrical conductivity. A magnification of 10,000× was performed. The accelerating voltage was 10 kV, and the average nanofiber diameter was calculated by measuring 100 random fibers and analyzing them using the Image J software for each nanofibrous scaffold.

#### 2.7.2. Average Pore Diameter

To analyze the average pore diameter, micrographs obtained by FE-SEM were processed with Image J software. The scale was adjusted, and then the image processing tool “Threshold” was used, and it was possible to establish the average pore diameter via intensity analysis.

#### 2.7.3. Stiffness (AFM)

An atomic force microscope (AFM) (MFP-30 Infinity, Oxford Instruments) was used for mechanical characterization. To determine these nanomechanical properties, we analyzed the Young’s modulus (E) of the nanofibers by means of the force–distance curves in the contact mode in air. The scaffolds were cut to 1 cm^2^ and placed on a 20 × 20 μm scanning grid. The tip of the silicon nitride (Si_3_N_4_) cantilever was placed on top of the nanofibers to perform uniaxial compression. The tip approach velocity was approximately 400 nm/s. The nanofibers were mapped at 20 points. The deflection of the cantilever tip was used as a feedback signal to maintain a constant force of 0.20 N/m over the scanned area. The force curves were fitted to the Hertz model to describe the elastic deformation of the nanofibers, and Hooke’s law was used to calculate the Young’s modulus. This methodology was developed as reported by Song et al. and Yilmaz et al. [28,29].

#### 2.7.4. Thermal Degradation

Thermogravimetric analysis (TGA) and differential scanning calorimetry (DSC) were used to measure changes in the mass loss and thermal behavior, respectively, of nanofiber scaffolds. Here, 8 mg of each sample was weighed on the analytical balance and placed in the cell of the SDT Q600-TA Instruments equipment. The parameters were set in a nitrogen atmosphere of 40 mL/min, and the analysis was performed in a temperature range of 28 °C to 700 °C at a heating rate of 10 °C/min.

#### 2.7.5. Chemical Interaction (FTIR)

The chemical interaction of the functional groups of the PVA/SF composite nanofibrous scaffolds was determined by an attenuated total reflectance Fourier transform infrared (ATR-FTIR) spectrophotometer (Shimadzu IR Trace-100 model). The ATR-FTIR spectrum of each scaffold was detected in the 4000 to 500 cm^−1^ wavenumbers, with 32 scans at a resolution of 4 cm^−1^ in transmittance mode, under ambient conditions.

#### 2.7.6. Wettability

The wettability or water contact angle (WCA) measurements were used to analyze the hydrophilicity of the scaffolds. The four kinds of scaffolds were measured using a DataPhysics OCA 15EC contact angle goniometer. Briefly, 20 μL of deionized water was dropped onto the surface of each scaffold. Then, the images of the droplets were captured using a high-resolution camera, and the angle generated between the droplet and the scaffold surface was analyzed. Data were taken in triplicate for each scaffold.

### 2.8. In Vitro Test

#### 2.8.1. MG-63 Cell Viability

To determine the viability of pure PVA nanofibers and PVA/fibroin/Ag-NPs with MG-63 cells, a CellTiter 96^®^ aqueous cell proliferation assay (MTS) was performed. Initially, human MG-63 cells were cultured in DMEM medium supplemented with 10% fetal bovine serum (FBS) in a cell culture flask at greater than 95% confluency and incubated at 37 °C in a CO_2_ atmosphere. The nanofibers were sterilized for 20 min per side with ultraviolet light in the CL-1000 Ultraviolet Crosslinker at 3000 J. The nanofibers were then washed twice with 1 mL of PBS and sterilized again by UV light. MG-63 cells at a concentration of 500,000 cells per well were grown on the PVA pristine and PVA/fibroin/Ag-NPs scaffolds. After 24 h of incubation, the 3-(4,5-dimethylthiazol-2-yl)-5-(3-carboxymethoxyphenyl)-2-(4-sulfophenyl)-2H-tetrazolium bromide (MTS) reagent was added in the presence of phenazine methosulfate (PMS). MTS and PMS (2:1 mg/mL) were prepared in sterile PBS, and 20 µL per well, with the light of the cabinet turned off as it is photosensitive, was added and incubated for 4 h before being taken to the plate reader of the BioTek spectrophotometer to estimate the percentage of cell viability from the absorbance at 490 nm.

#### 2.8.2. Alizarin Red (ARS)

Alizarin red (ARS) is a dye used in biochemical assays to quantitatively determine by colorimetry the presence of calcium deposits in the cells of an osteogenic cell line. For this assay, PVA nanofiber scaffolds were cut and subjected to a UV sterilization process in the Crosslinker CL-1000 Ultraviolet at 3000 joules for 20 min. This was followed by two washes with PBS and UV sterilization again. Subsequently, 40,000 viable MG-63 cells were plated onto the membranes in a 96-well plate, bringing the final volume to 100 µL of DMEM medium supplemented with 10% fetal bovine serum (FBS). Mineralization (calcification by MG-63 cells) was assessed on the membranes at days 3, 7, and 10. After this time, the cells were fixed with 10% formaldehyde and stored in a refrigerator at 4 °C for 6 h, then the formaldehyde was removed and stained with 100 µL of 1% alizarin red-S (ARS) solution (Sigma Aldrich, Saint Louis, MO, USA) (pH 4.2) in each well. They were incubated for 20 min at room temperature on an orbital shaker. The alizarin red solution was then removed, and the wells were washed three times with distilled water. The plate was allowed to dry, and finally, it was washed with 10% acetic acid to dissolve the dye. The absorbance of each well was measured at 405 nm using an iMark^®^ Microplate Absorbance Reader ELISA microplate reader (BioRad, Hercules, CA, USA). The results represent the average and standard deviation of three assays. On day 10, the extent of staining (mineralization) was observed under an inverted light microscope (Motic AE31, Barcelona, Spain).

#### 2.8.3. Alkaline Phosphatase (ALP)

Alkaline phosphatase (ALP) is the initial differentiation marker of osteoblasts, which decreases with age when bone growth has consolidated. ALP is expressed in mineralized tissue cells as well as reducing the concentration of extracellular pyrophosphate, an inhibitor of mineral formation. It plays a key role in bone calcification. PVA nanofiber scaffolds were sterilized in UV light at 3000 J for 20 min per side, and membranes were washed twice with PBS and sterilized again in UV light. Then, 10,000 MG-63 cells were seeded per well, and brought to a final volume of 100 µL of supplemented DMEM medium (SFB 10%).

After 24 h of incubation with the treatment, the culture medium was removed with a pipette and washed with 100 µL of PBS 1X. For 30 min, 100 µL of 5% formaldehyde was added, followed by two washes with 100 µL of PBS 1X, and lysed with 100 µL of 0.1% Triton with 0.1% sodium citrate for 2 min. A wash with 100 µL of 1X PBS was performed again. Then, 0.1 M Tris-HCl pH 7.5, 3% BSA, and 10% fetal bovine serum were added for 1 h. Here, 100 µL of primary antibody (ALP, Santa Cruz Biotechnology, Catalog number SC-398461) (1:1000) was incubated for 12 h in the refrigerator. A wash with 100 µL of PBS 1X was performed. Then, 100 µL of secondary antibody (Goat Anti-Mouse Igg, (H&L), peroxidase conjugated, Santa Cruz Biotechnology, Catalog Number 31430 PIERCE) (1:1000) was added (1:1000) and incubated for 12 h in the refrigerator. Two washes were performed with 100 µL of 1X PBS, then 50 µL of TMB substrate was added, incubated for 5 min, and 0.16 M formic acid (H_2_SO_4_) was added to stop the reaction. The plate was read at an absorbance of 450 nm on the Varioskan LUX ELISA reader.

#### 2.8.4. Statistical Analysis

Cellular experiments (cell viability, alizarin red, alkaline phosphatase) were performed in triplicate for each concentration, and all data were presented as mean ± standard deviation (SD), and an ordinary one-way ANOVA was used, followed by Tukey’s multiple comparisons test. A value of *p* < 0.05 was considered statistically significant in this study.

## 3. Results and Discussion

### 3.1. Characterization of Silver Nanoparticles (Ag-NPs)

#### 3.1.1. Formation of Ag-NPs (UV-Vis) Spectrometry

**Figure 1:**
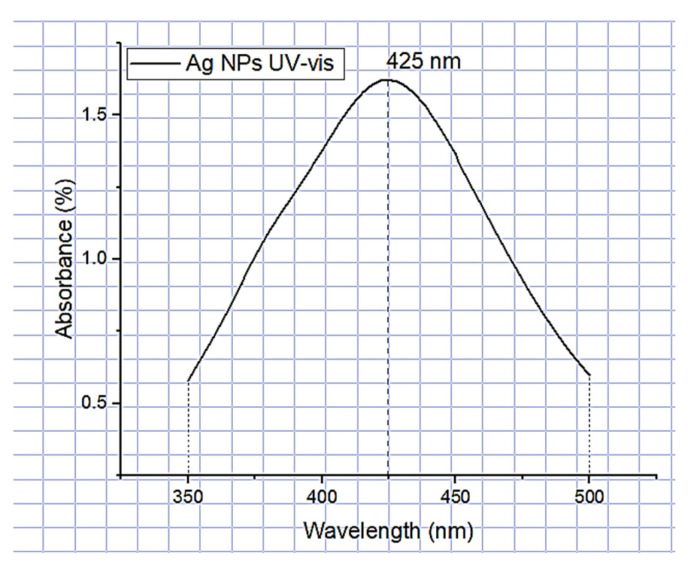
UV-vis of Ag-NPs.

UV-vis spectrometry allowed the analysis of the spectra obtained from the silver nanoparticle suspensions (Ag-NPs). The peak of the UV-vis absorption spectrum was located in the surface plasmon resonance absorption band (SPR) in the range of 350 to 500 nm, with a maximum absorption band peak at approximately 425 nm (Figure 1), indicating the formation of Ag-NPs. These results are similar to those obtained by Urena-Saborio et al. (425 nm) [20], Tran et al. (430 nm) [30], and Du et al. (435 nm) [31].

#### 3.1.2. Average Ag-NPs’ Size (DLS)

**Figure 2:**
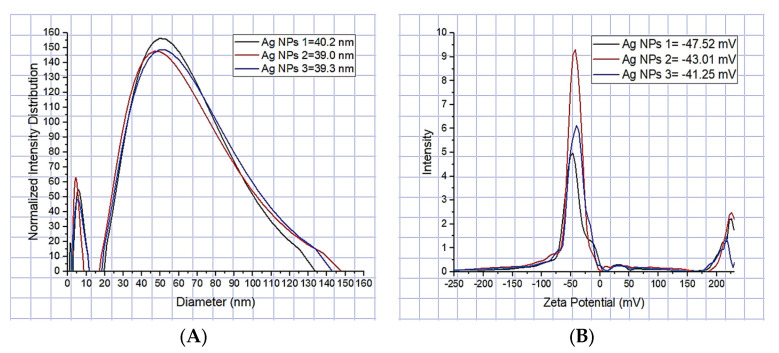
(**A**) Average diameter of Ag-NPs. (**B**) Z-Potential of Ag-NPs.

Dynamic light scattering (DLS) allowed determining the average size of silver nanoparticles of approximately 39.5 nm, based on consecutive measurements of nanoparticle subgroups, similar to that reported by J. Zapata-Giraldo et al. of 36 nm [32], with a variation of silver nanoparticle sizes from 1 to 138 nm (Figure 2A). The polydispersity index was 0.230, similar to that reported by Ballesteros et al. of 0.281 ± 0.073 [21]. Values close to 0 indicate that the sample is monodisperse, while values close to 1 indicate that the sample presents a great variety of sizes (polydisperse), which indicates that the Ag-NPs’ suspension was stable in nanoparticle sizes (monodisperse). The Z-potential was used to evaluate the surface charge of silver nanoparticles in colloidal suspension, which is important to evaluate their interaction with electrospun nanofibers. The Z-potential of the Ag-NPs was high, with a negative charge of −43.93 mV (Figure 2B). As reported by Mahajan et al., the Z-potential of ±40 to ±60 mV indicates that they are electrically stable [33]. Furthermore, this value shows that they are uniformly dispersed nanoparticles in the suspension, and due to this, they resist agglomeration.

#### 3.1.3. Morphology and Size of Ag-NPs (TEM)

**Figure 3:**
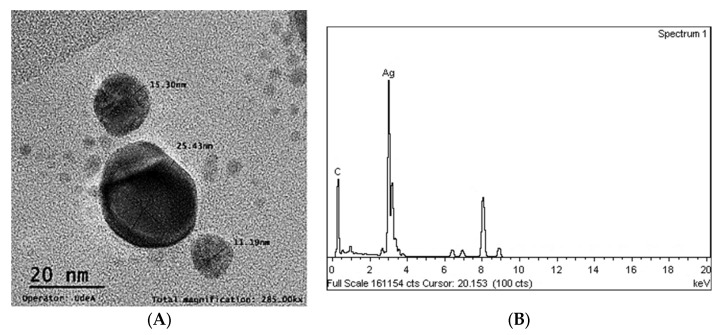
(**A**) TEM micrograph of Ag-NPs. (**B**) EDX spectra.

The shape of the silver nanoparticles was almost irregularly spherical, with an average size that ranged from 4.67 nm to 53.31 nm (Figure 3A). These average sizes are very similar to those obtained by DLS at 39.5 nm, and similar to Gallo Ramirez et al., 5 to 40 nm [34], and Zhang et al., 5–50 nm [35]. Trisodium citrate served the function of a reducing agent for silver nitrate and acted as a stabilizer since no significant agglomerations, which can be observed as darker nanoparticles in the gray scale, were present in the TEM micrographs. The EDX spectrum showed a peak at 3 keV that confirmed the presence of the silver element (Ag) in the composition of the nanoparticles (Figure 3B). The EDX spectrum does not have a significant presence of oxygen, so it is interpreted that the silver is not oxidized, similar to what was reported by Srivastava et al. [8] and by Gallo Ramírez et al. [34].

### 3.2. Characterization of Nanofiber Scaffolds

#### 3.2.1. Morphology of the Electrospun Nanofibers

**Figure 4:**
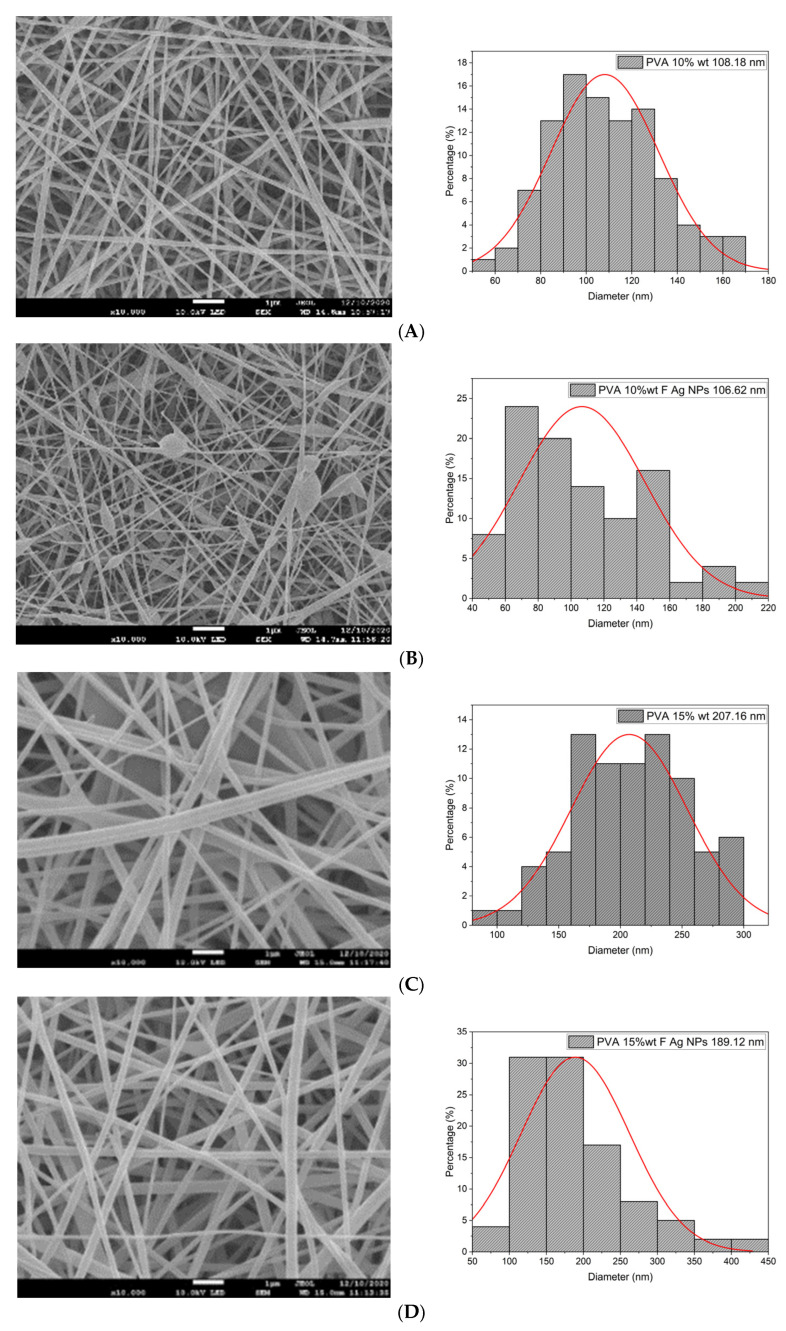

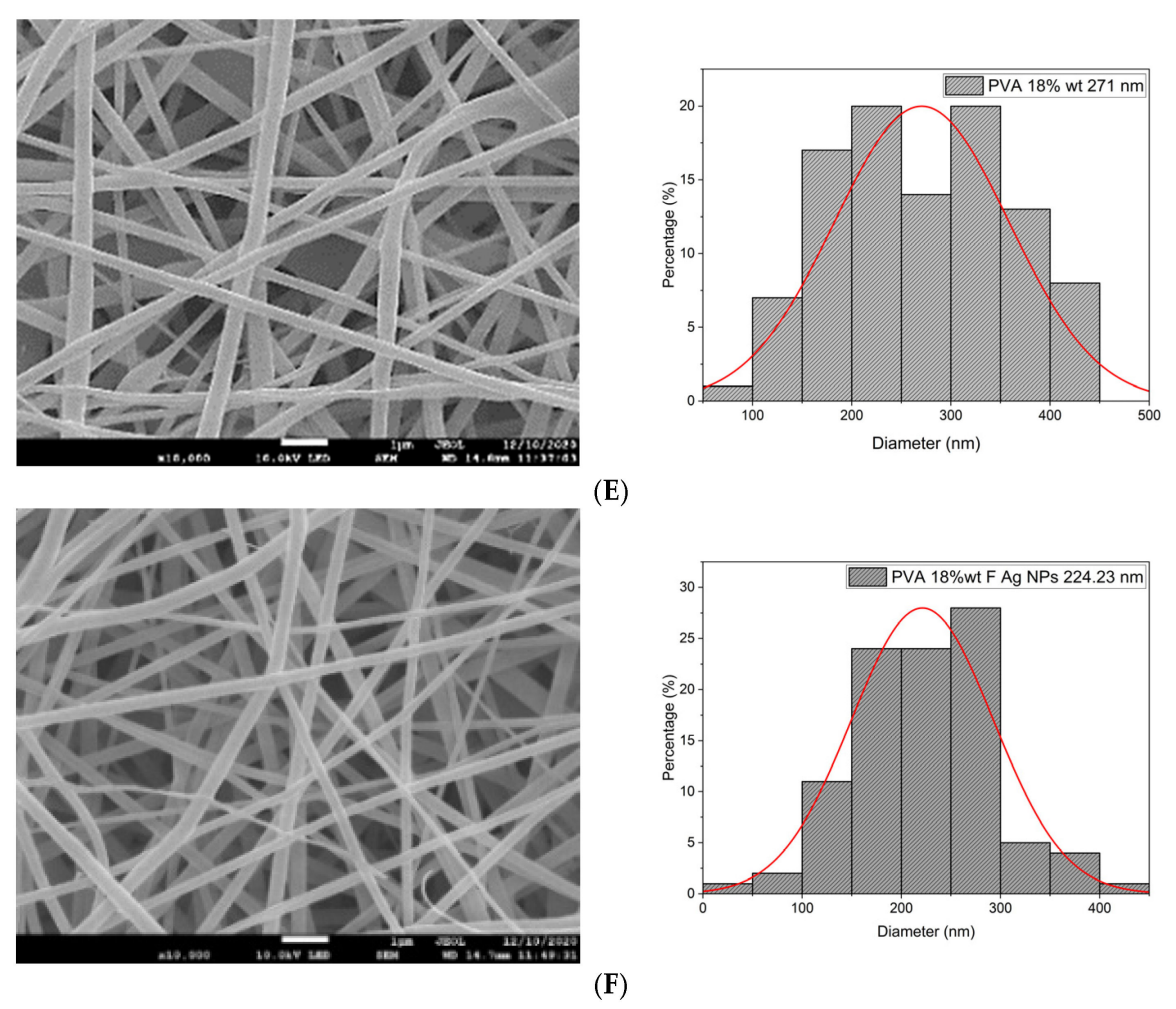
SEM images and average nanofiber diameter histograms of the (**A**) PVA 10% wt, (**B**) PVA10/SF/Ag-NPs, (**C**) PVA 15% wt, (**D**) PVA15/SF/Ag-NPs, (**E**) PVA 18% wt, and (**F**) PVA18/SF/Ag-NPs.

In this work, PVA pristine and PVA/SF/Ag-NPs composite scaffolds were produced with randomly oriented fibers, and a smooth and uniform morphology under controlled parameters. The average nanofiber diameter of PVA 10% wt pristine was 108.18 nm (Figure 4A), and this decreased with fibroin and Ag-NPs to 106.62 nm (Figure 4B). No significant changes were noted in the mean diameter. However, there were changes in their morphology with the addition of fibroin and Ag-NPs, causing the formation of bead defects because they reduced the viscosity of the solution. PVA 15% wt pristine scaffolds had an average diameter of 207.16 nm (Figure 4C), and their diameter increased to 227 nm with PVA 18% wt pristine (Figure 4E). The increase in the PVA solution concentration increased the average diameter of the nanofibers, which is similar to that reported by Jin et al., who also electrospun PVA of medium molecular weight (89,000–98,000 Da) at 12% and obtained an average diameter of 170 nm [36]. PVA15/SF/Ag-NPs had an average diameter of 189.12 nm (Figure 4D), which increased to PVA18/SF/Ag-NPs at 224.23 nm (Figure 4F). However, the addition of fibroin and silver nanoparticles had an effect on reducing the average fiber diameter compared to pure PVA scaffolds. Figure 4 presents the morphology of different PVA electrospun nanofiber mats and their respective histograms.

#### 3.2.2. Average Pore Diameter

Porosity allows oxygenation, vascularization processes, nutrient filtration, cell migration, adhesion, and proliferation, all necessary for bone regeneration. Optimal pore sizes for bone regeneration are in the range of 100 to 500 μm [37]. The PVA 10% wt pristine scaffold exhibited an average pore diameter of 65.44 μm and increased in PVA10/SF/Ag-NPs to 252.29 μm, and this is an optimal pore size to allow bone regeneration. PVA 15% wt pristine had an average pore size of 65.84 μm and decreased in PVA15/SF/Ag-NPs to 62.43 μm. PVA 18% wt pristine had an average pore size of 63.63 μm and decreased in PVA18/SF/Ag-NPs to 62.55 μm. All pore sizes were larger than 40 μm, indicating that there were interconnections between them [38]. They were also larger in size than those reported in the literature for PVA (Mw: 20,000 Da) at 11% wt, 4.98 ± 0.3 μm, also measured using Image J software for image processing [39], indicating that the molecular weight has an influence on the average pore size. Giovanni et al. reported a pore size of 309.49 ± 161.85 nm for pure PVA membranes and a reduced size of 49.59 ± 18.48 nm for PVA/SF (70:30), measured by image processing in Image J software [7].

#### 3.2.3. Stiffness (AFM)

By means of the 20 nano-indentations performed by AFM on the nanofibers, the nanomechanical properties (Young’s modulus, *E*) could be determined using the Hertz model to describe the elastic deformation of the nanofibers, and Hooke’s law was used to calculate the average Young’s modulus for each of the concentrations of PVA. The stiffer a material is, the higher its elastic modulus, or Young’s modulus (*E*). The stiffness of nanofibers can have a potential impact on the behavior of bone cells as a result of their interconnected structure. Due to the protruding structure of the nanofibers, the AFM topographic images presented high relief, so it was not possible to evaluate the roughness, which is related to increased cell adhesion and proliferation and produces osseointegration [40,41]. Nanofiber scaffolds, when implanted in vivo, increase the Young’s modulus over time in the process of osseointegration, resembling the stiffness of native bone.

**Figure 5:**
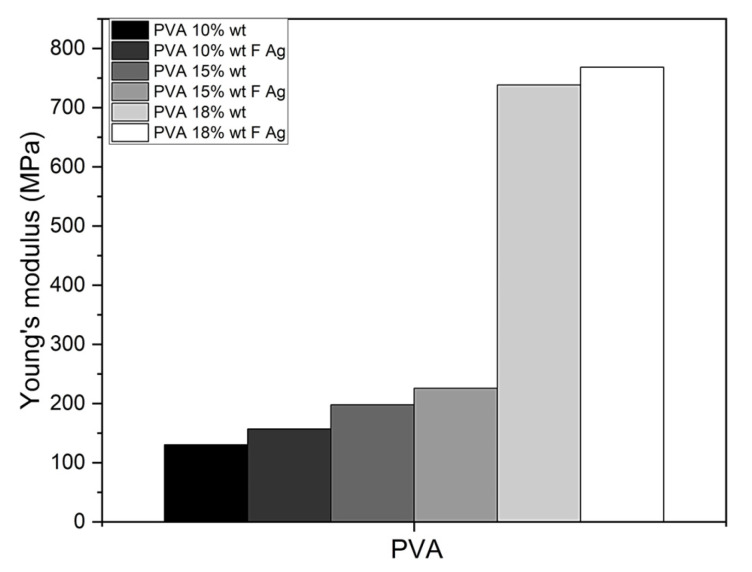
Young’s modulus (*E*) of PVA membranes.

PVA 10% wt pristine obtained an average Young’s modulus of 130.27 MPa and increased by 17% in PVA 10% wt/SF/Ag-NPs to 156.94 MPa. PVA 15% wt pristine exhibited an elastic modulus of 197.86 MPa and increased by 12.35% in PVA 15% wt/SF/Ag-NPs to 225.72 MPa. PVA 18% wt pristine showed a Young’s modulus of 738.20 MPa and increased by 4% in PVA 18% wt/SF/Ag-NPs to 768.38 MPa (Figure 5). As can be seen, the addition of fibroin and silver nanoparticles had an influence on increasing the stiffness of PVA membranes. The fibroin provides stiffness by having greater mechanical strength, while the silver nanoparticles act as stiffening agents. Furthermore, increasing the PVA concentration increased the Young’s modulus (*E*). In other investigations, they have reported a lower Young’s modulus of 0.83 MPa in 10% wt PVA membranes (Mw: 205,000 g/mol) [28], 0.826 MPa in 10% wt PVA membranes (Mw: 205,000 g/mol) [14], and 16.64 MPa in 10.71% wt PVA membranes (Mw: 85,000–124,000 g/mol) [42]. Enayati et al. reported a higher Young’s modulus of 1.27 ± 0.28 GPa in PVA 8% wt (Mw: 124,000 g/mol) [43]. PVA membranes produced in the present study comply with a Young’s modulus (0.13–0.768 GPa) similar to that of cancellous bone (0.1–2 GPa) [44], so they could work well as bone substitutes.

#### 3.2.4. Thermal Degradation

##### Mass Loss (TGA)

**Figure 6:**
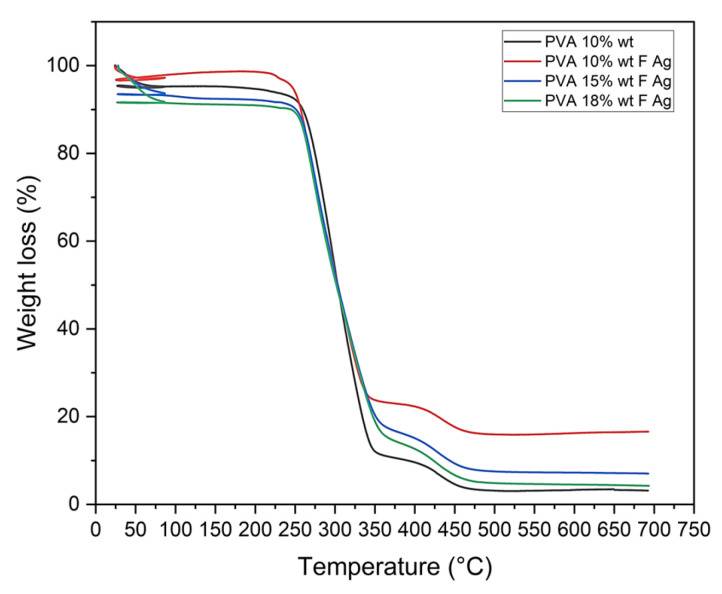
TGA of PVA 10% wt pristine and PVA 10%, 15%, and 18% wt/SF/AgNPs composite scaffolds.

Thermogravimetric analysis (TGA) of PVA pristine and PVA/fibroin/Ag-NPs composite scaffolds exhibited 3 regions of weight loss: Region I (24–80 °C) recorded a weight loss of 4.8% for PVA 10% wt, 2.67% for PVA 10/SF/Ag-NPs, 9.64% for PVA 15/SF/Ag-NPs, and 8.54% for PVA 18/SF/Ag-NPs (attributed to the removal of water residues). Region II (80–350 °C) showed a weight loss between 72% and 82% (related to the decomposition of the PVA side chain), and region III (350–500 °C) demonstrated a weight loss between 7% and 11% (related to the decomposition of the PVA main chain), and a residual weight between 3% and 16%. The addition of fibroin and silver nanoparticles improved the thermal stability of PVA membranes (Figure 6). These results are similar to Chahal et al., who fabricated hydroxyethyl cellulose HEC/PVA nanofibers for bone tissue engineering [39], and Singh et al., who developed PVA/fibroin/bioglass sol gel scaffolds that allowed to provide higher thermal stability at the temperature of 700 °C while retaining a residual of 10–11% compared to completely consumed PVA/fibroin [45]. Islam et al. improved the thermal stability of pullulan/PVA/4% wt silver nanoparticle composite nanofibers with a residual of about 25° at 600 °C [46].

##### Thermal Behavior (DSC)

**Figure 7:**
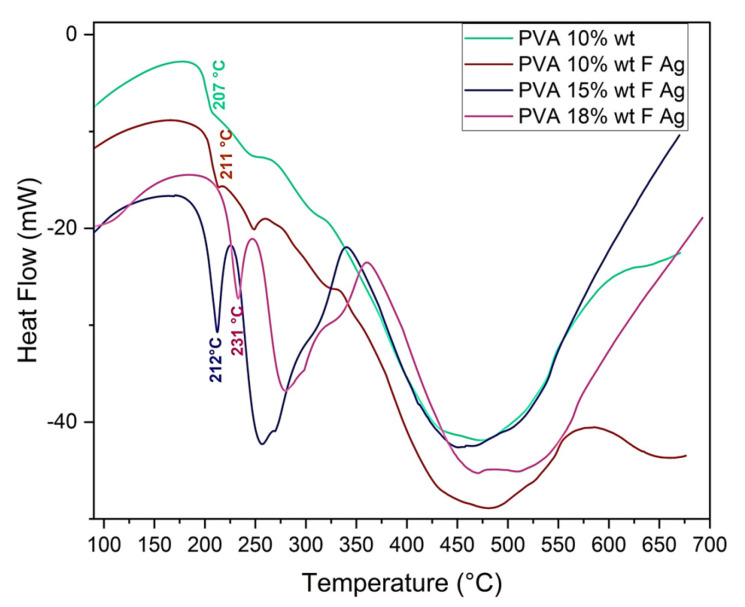
DSC of PVA 10% wt pristine and PVA 10%, 15%, and 18% wt/SF/Ag-NPs composite scaffolds.

PVA pristine and PVA/fibroin/Ag-NPs composite scaffolds were analyzed by differential scanning calorimetry (DSC), and they presented endothermic peaks. The former was related to moisture evaporation, and the latter peaks could be attributed to the thermal degradation of PVA, with melting temperatures (Tm) of PVA 10% wt pristine at 207 °C, PVA 10/SF/Ag-NPs at 211 °C, PVA 15/SF/Ag-NPs at 212 °C, and PVA 18/SF/Ag-NPs at 231 °C. The melting temperature required to degrade the amorphous PVA chains increased with the increasing PVA concentration (Figure 7). The endothermic PVA peak became sharper and shifted, indicating interactions between fibroin and PVA. These results were similar to those of Kalani et al., of 220 °C [1], Kaur et al., of 222–226 °C [47,48], Shao et al., of 200 °C [49], and Lee et al., of 296 °C for PVA and of 290 °C for PVA/SF [50].

#### 3.2.5. Chemical Interaction (FTIR) Spectroscopy

**Figure 8:**
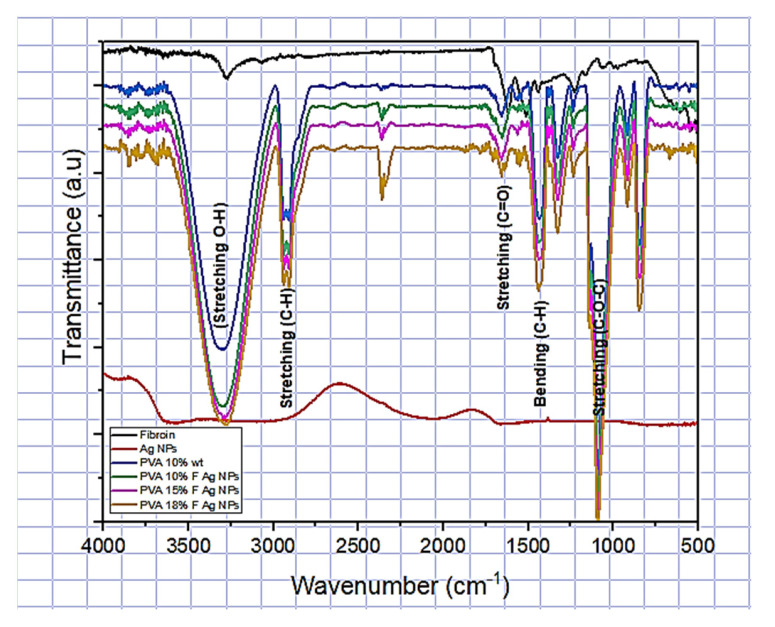
ATR-FTIR spectra of PVA pristine and PVA/SF/Ag-NPs composite scaffolds.

The FTIR spectroscopy analysis in ATR mode confirmed the blending of PVA, fibroin, and Ag-NPs in composite scaffolds and demonstrated good interactions between them, caused by strong intermolecular hydrogen bonds. As can be seen in Figure 8, for PVA 10% wt pristine, the characteristic peaks were observed at 3500–3000 cm^−1^ (OH stretches from the intermolecular and intramolecular hydrogen bonds), 2930 cm^−1^ (aliphatic C-H stretching), 1656 cm^−1^ (C=O stretching), 1440 cm^−1^ (C- H_2_ bending), 1085 cm^−1^ (C-O-C stretching), and 860 cm^−1^ (amorphous C-O stretching). There was an increase in the intensity of the 2360 cm^−1^ peak related to the increase in the PVA concentration. At 1650 cm^−1^ (amide I C=O stretching), fibroin displayed the characteristic peaks of the amorphous silk I-helix conformation. At 1560 cm^−1^, amide II secondary N-H bending demonstrates the existence of a β-sheet structure, and at 1238 cm^−1^, amide III C-N and N-H [1,2,9,11]. In relation to silver nanoparticles, the peak at 1277 cm^−1^ related to silver nitrate (NO_3_^−^) was not observed, which indicates that pure nanoparticles were obtained, and their reduction was achieved, and this corresponds to what was reported by Patil et al. [26].

#### 3.2.6. Wettability

PVA and fibroin are of a hydrophilic nature. In the range of 40–70°, it has been reported that an optimal water contact angle (WCA) is necessary for cell attachment and proliferation. Hydrophilic surfaces can absorb more serum and growth factors from the medium, which contributes to enhanced osteoblast cell culture [1,51]. Bhattacharjee et al. reported that the WCA of the PVA/SF nanofiber scaffolds decreased with an increase in the silk fibroin concentration, increasing the hydrophilicity [22]. Rama et al. also indicated a reduction in the contact angle after the addition of fibroin [2]. Others have reported that β-sheets, in the structure of *Bombyx mori* fibroin, consist of repetitive hydrophobic domains of amino acids and increase the WCA of PVA/SF scaffolds [1,7,9]. Giovanni et al. and Sayed et al. indicated an increase in WCA in PVA/SF scaffolds due to heat crosslinking and immersion of the membranes in methanol [7] and glutaraldehyde [9].

In this study, the WCA of PVA 10% wt, 15% wt, and 18% wt pristine scaffolds were 42.5°, 45.1°, and 42°, respectively, similar to the previously reported 42° [7] and 41.16° [9]. The WCA of PVA composite scaffolds decreased with the incorporation of fibroin and silver nanoparticles to PVA10/SF/Ag-NPs at 31.1°, PVA15/SF/Ag-NPs at 32°, and PVA18/SF/Ag-NPs at 31°. The PVA concentration had no influence on the hydrophilic characteristics, as shown in Table 2.

### 3.3. In Vitro Test

#### 3.3.1. MG-63 Cell Viability

In the PVA micrographs taken under an inverted optical microscope, it can be observed that, as time passed from 10 min to 36 h, MG-63 cells were adhering to the surface of the PVA membranes. In the case of PVA18/fibroin/Ag-NPs at 36 h, the number of cells exceeded the number of cells with respect to the control (Table 3).

**Figure 9:**
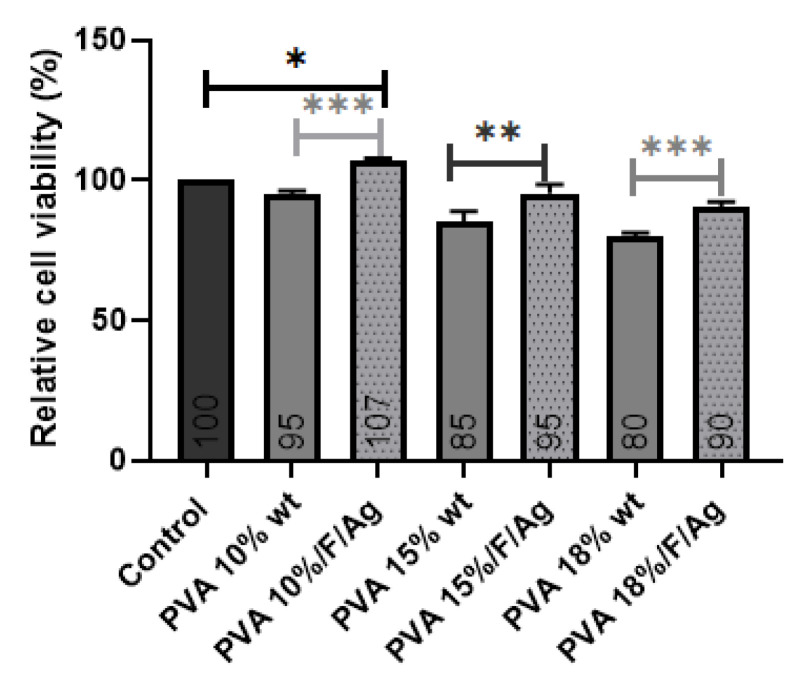
MTS test of percentage of relative MG-63 cell viability for pure PVA and PVA/fibroin/Ag-NPs composite membranes. Statistical analysis was developed with ordinary one-way ANOVA followed by Tukey’s multiple comparisons test, *p* < 0.05. * Low significant difference, ** Significant difference, *** high significant difference.

PVA 10% wt/fibroin/Ag-NPs composite scaffolds exhibited a relative cell viability percentage of 107%, and there was a little significant difference with respect to the control (cells without treatment). All samples were measured after 24 h of culture with MG-63 cells, and the viability for all samples was higher than 70% (Figure 9), which according to ISO 10993-5 indicates that they are not cytotoxic since there was a high cell survival rate. In PVA pristine, increasing the concentration from 10% to 18% wt reduced viability, indicating that this material alone is not sufficiently bioactive. The addition of fibroin and silver nanoparticles showed a significant difference in improving MG-63 cell viability and the interactions with the scaffolds.

#### 3.3.2. Alizarin Red (ARS)

**Figure 10:**
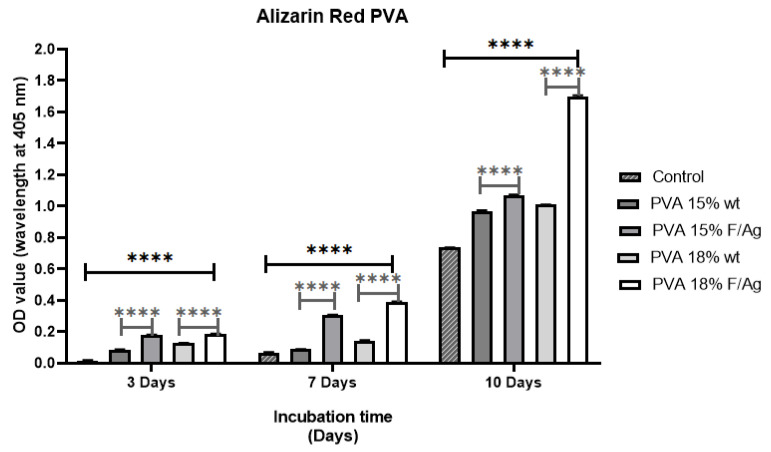
Alizarin red test for pure PVA and PVA/fibroin/Ag-NPs composite membranes on days 3, 7, and 10. Statistical analysis was developed with ordinary one-way ANOVA followed by Tukey’s multiple comparisons test, *p* < 0.05. **** high significant difference.

The osteogenic potential of the PVA nanofiber scaffolds was evaluated by measuring the biomineralization capacity (calcium deposition) using alizarin red (ARS), which is a colorimetric assay. Staining showed calcium deposition with respect to the control at 3, 7, and 10 days. The results were qualitatively analyzed with optical microscope images and quantitatively by absorbance (Figure 10). From the micrographs obtained on day 10, it was possible to determine the formation of the mineralized matrix in the PVA membranes cultured with MG-63 osteoblasts (Table 4), which is an important phase for the formation of the extracellular matrix of bone. As expected, the control showed lower calcium deposition after 10 days, and there was a significant increase in the mineralization of PVA18/fibroin/Ag-NPs composite scaffolds (red nodules) compared to PVA 18% wt pristine and the control.

As a result, the addition of fibroin and silver nanoparticles to PVA scaffolds made them suitable as substrates for bone tissue engineering, similar to the findings of Kalani et al., who found that hADSCs cells produced more calcium on Rosuvastatin (RSV)-loaded nanofibers than the blank on day 14 and significantly higher (*p* < 0.01) on day 21 of culture [1]. Bhattacharjee et al. demonstrated that MG-63 cells had homogeneous mineralization on scaffolds containing PVA/fibroin (*Antheraea mylitta*) at 2% and 4% wt, in contrast to PVA pure nanofibers, which only showed patches on day 21 of culture [22]. Singh et al. indicated that there was a significantly (*p* < 0.05) higher degree of mineralization (calcium nodule formation) in MSCs seeded in SF/PVA/bioglass than in SF/PVA at day 14 of culture [45].

#### 3.3.3. Alkaline Phosphatase (ALP)

Alkaline phosphatase (ALP) is an enzyme used as an early quantitative marker associated with osteogenic differentiation, which aids new bone formation [1,45,47]. The ALP level decreases with age: an adolescent has a higher ALP level than an adult whose bone growth has terminated [52]. Alkaline phosphatase plays an important role in extracellular matrix mineralization through cleavage of the organic phosphate ester, leading to calcium phosphate formation [45].

**Figure 11:**
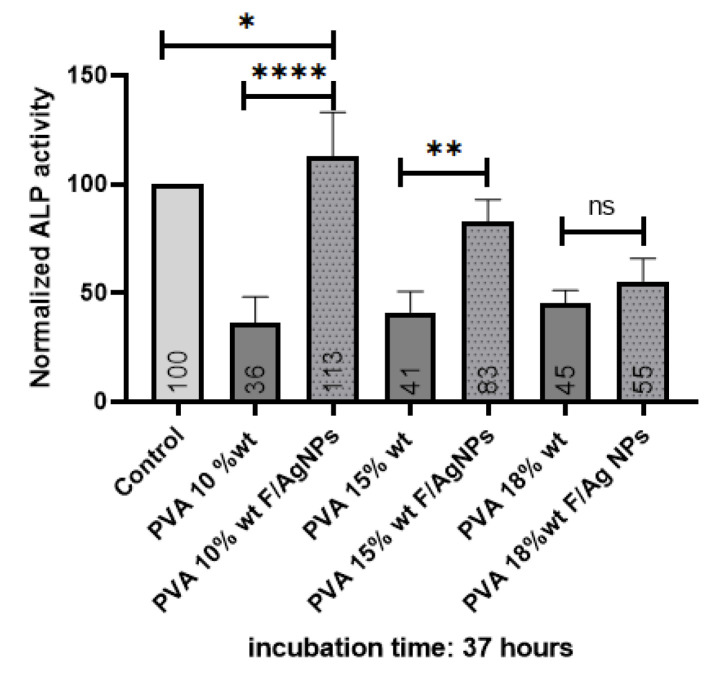
Alkaline phosphatase of MG-63 cells after 37 h of culture with PVA membranes. Statistical analysis was developed with ordinary one-way ANOVA followed by Tukey’s multiple comparisons test, *p* < 0.05. * Low significant difference, ** Significant difference, **** High significant difference, ns No significant difference.

After 37 h of culture, the PVA10/fibroin/Ag-NPs composite scaffolds presented significantly higher alkaline phosphatase (ALP) expression than PVA 10% wt pristine and the control. This increase could be due to the higher surface roughness, which thus promoted adhesion and proliferation of MG-63 cells to the membranes, providing nucleation sites for mineral deposition, leading to increased extracellular matrix production and tensile strength. PVA scaffolds with fibroin and silver nanoparticles favored the differentiation of seeded MG-63 osteosarcoma cells, thanks to the hydrophilic nature of PVA and fibroin (Figure 11). These results are similar to those reported in [3,47].

PVA 18% wt/fibroin/Ag-NPs were selected as a scaffold for the growth of MG-63 cells in bone tissue engineering since they presented the best nanofiber formation with a morphology without droplet defects and an average diameter of 224.23 nm, with interconnected pores with an average diameter of 62.55 μm, which favored cell infiltration. It was the scaffold with the highest stiffness of 0.768 GPa, similar to cancellous bone of 0.1–2 GPa. Although the human temperature is 37 °C, we wanted to evaluate the thermal behavior of the scaffolds due to the higher concentration of PVA, meaning the higher the melting temperature required to degrade the amorphous chains. There was a good interaction between the functional groups of the PVA/fibroin/Ag-NPs confirmed by FTIR, and there was an increase in the intensity of the peak at 2360 cm^−1^, as the PVA concentration increased. Although, the PVA 18% wt/fibroin/Ag-NPs membranes were very hydrophilic (31°), they allowed good cell attachment, which was confirmed at 36 h of the viability test, where these scaffolds exceeded the number of cells with respect to the control. Mineralization was also higher after 10 days of culture, confirmed by the alizarin red test. No significant difference was obtained in the alkaline phosphatase test for the PVA 18% wt/fibroin/Ag-NPs scaffolds, which could be due to the fact that the measurement was after 37 h of culture. A longer period of analysis between 14 and 21 days is recommended in future work to determine early osteogenic differentiation.

## 4. Conclusions

In this study, PVA pristine and PVA/*Bombyx mori* fibroin/silver nanoparticles (Ag-NPs) composite nanofiber scaffolds were prepared by electrospinning. UV-vis spectrometry was used to investigate the formation of silver nanoparticles related to the peak of the absorption spectrum at 425 nm, with an average size of 39.5 nm in a monodisperse colloidal suspension that was electrically stable and irregularly spherical. The average diameter of nanofibers decreased with the addition of fibroin and Ag-NPs, causing the formation of bead defects because they reduced the viscosity of the solution. PVA 10% wt/SF/Ag-NPs exhibited an average pore size of 252.29 μm, which is optimal to allow bone regeneration. The addition of fibroin and silver nanoparticles had an influence on increasing the stiffness of PVA membranes; furthermore, at higher PVA concentrations, this also increased the Young’s modulus (*E*). PVA 18% wt/SF/Ag-NPs showed the highest stiffness of 768.38 MPa, so this could work well as a bone substrate. PVA 10% wt/SF/Ag-NPs was the scaffold with the lowest mass loss, with a residual weight of 16.58% at 700 °C. PVA 18% wt/SF/Ag-NPs had a melting temperature (Tm) of 231 °C, and the higher the PVA concentration, the higher the melting temperature needed to degrade the amorphous PVA chains. FTIR allowed to observe good chemical interactions between the functional groups of the PVA/SF/Ag-NPs composite nanofibrous scaffolds. The WCA of PVA composite scaffolds decreased with the incorporation of fibroin and silver nanoparticles. In future work, it is recommended to increase the contact angle to between 40° and 70° to allow better cellular attachment and better performance as a substrate for cell growth. At 36 h of culture, the number of MG-63 cells seeded on the PVA18% wt/SF/Ag-NPs scaffolds exceeded the control. All samples had a viability higher than 70%, demonstrating no cytotoxicity according to ISO 10993-5. At 10 days of culture with MG-63, the PVA 18% wt/SF/Ag-NPs composite scaffolds’ mineralization increased compared to the PVA 18% wt pristine and the control, as verified by the alizarin red assay. After 37 h of culture, the PVA 10% wt/fibroin/Ag-NPs composite scaffolds had significantly higher alkaline phosphatase (ALP) expression than the PVA 10% wt pristine and the control, indicating early osteogenic differentiation.

## Figures and Tables

**Table 1:** Electrospinning solutions.

Solution	PVA	Fibroin	Ag-NPs
PVA 10% wt	10% wt	--	--
PVA10/SF/Ag-NPs	10% wt	2% wt	0.5% wt
PVA 15% wt	15% wt	--	--
PVA15/SF/Ag-NPs	15% wt	2% wt	0.5% wt
PVA 18% wt	18% wt	--	--
PVA18/SF/Ag-NPs	18% wt	2% wt	0.5% wt

**Table 2:** Water contact angle of pristine PVA scaffolds and PVA/fibroin/Ag-NPs composites.

**PVA 10% wt**	**PVA 15% wt**	**PVA 18% wt**
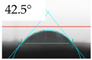	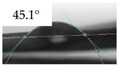	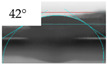
**PVA10/SF/Ag-NPs**	**PVA15/SF/Ag-NPs**	**PVA18/SF/Ag-NPs**
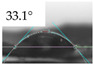	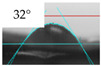	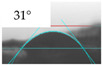

**Table 3:** Optical micrographs of MG-63 cells in contact with PVA membranes at times of 10 min, 24 h, and 36 h.

Time	PVA 10% wt	PVA10/SF/Ag-NPs	PVA 15% wt	PVA15/SF/Ag-NPs	PVA 18% wt	PVA18/SF/Ag-NPs	Control
10 min	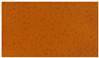	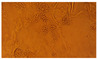	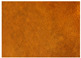	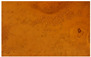	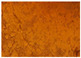	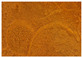	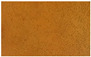
24 h	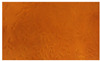	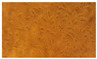	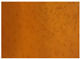	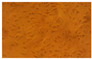	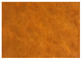	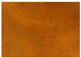	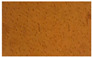
36 h	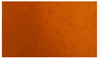	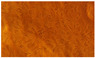	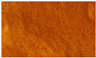	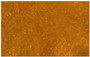	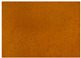	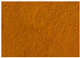	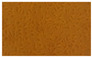

**Table 4:** Optical micrographs of calcium deposition on PVA membranes cultured with MG-63 cells, with respect to the control on day 10.

**PVA 15% wt**	**PVA 15% F Ag-NPs**	**Control**
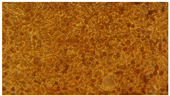	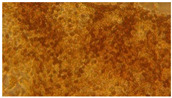	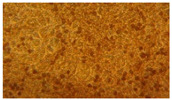
**PVA 18% wt**	**PVA 18% F Ag-NPs**	
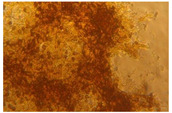	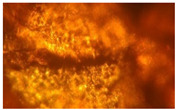	

## Data Availability

PhD thesis “Nanostructured membranes of polymer plus fibroin and silver nanoparticles constructed by electrospinning technique with potential use as a bone substitute when stimulated by external electric fields” Metropolitan Technological Institute (ITM). Characterization of electrospun silk fibroin scaffolds for bone tissue engineering: A review. https://revistas.itm.edu.co/index.php/tecnologicas/article/view/1573; Poly (vinyl alcohol)/Silk Fibroin/Ag NPs composite nanofibers for bone tissue engineering. https://ieeexplore.ieee.org/abstract/document/9629992.
